# Effects of Protocatechuic Acid on Endothelial Function and HDL Functionality in Hypercholesterolemic Subjects: A Randomized Double-Blind Placebo-Controlled Trial

**DOI:** 10.3390/nu18183056

**Published:** 2026-09-18

**Authors:** Guanyu Chen, Yihan Wang, Qiuhui Xu, Ruxun Zhao, Yihao Yuan, Dongliang Wang

**Affiliations:** 1 Department of Nutrition, School of Public Health, Sun Yat-sen University (Northern Campus), Guangzhou 510080, China; chengy268@alumni.sysu.edu.cn (G.C.); wangyh266@mail2.sysu.edu.cn (Y.W.); xuqh36@mail2.sysu.edu.cn (Q.X.); zhaorx9@mail2.sysu.edu.cn (R.Z.); yuanyh55@mail2.sysu.edu.cn (Y.Y.); 2 Guangdong Engineering Technology Center of Nutrition Transformation, Guangzhou 510080, China

**Keywords:** protocatechuic acid, endothelial function, HDL functionality, endothelial nitric oxide synthase activity, HDL subclass distribution

## Abstract

**Background**: High-density lipoprotein (HDL) exerts atheroprotective properties, partially by maintaining endothelial function. Protocatechuic acid (PCA) has been shown to improve endothelial function in mice. However, whether PCA improves endothelial function in humans is currently unknown. Our studies aimed to evaluate the effects of PCA on endothelial function and HDL concentration, subclass distribution, and functionality in humans. **Methods**: A double-blind, randomized, placebo-controlled trial was conducted in 150 hypercholesterolemic participants (aged 35–65 y) daily receiving 20 mg PCA or placebo capsules for 12 weeks. Endothelial function [brachial artery flow-mediated dilation (FMD)], serum cyclic guanosine monophosphate (cGMP), HDL concentration, subclass distribution (pre-β-HDL, HDL_3_, HDL_2_), and functionality [endothelial nitric oxide synthase (eNOS) activity] were tested at baseline, 6 weeks, and 12 weeks. **Results**: PCA consumption for 12 weeks significantly improved brachial artery FMD [1.77% (95% CI: 1.46 to 2.08)] and increased cGMP [11.72 pmol/L (95% CI: 6.16 to 17.28)] as compared to the placebo treatment, along with increased HDL_3_, decreased HDL_2_, and non-significantly changed in HDL and pre-β-HDL. PCA consumption also increased eNOS activity of HDL [0.39 (95% CI: 0.29 to 0.49)]. In the PCA group, the changes in HDL functionality on eNOS activity were positively correlated with the changes in brachial artery FMD, cGMP, and HDL_3_, whereas negatively correlated with HDL_2_. **Conclusions**: PCA consumption improves endothelial function and HDL functionality in humans with hypercholesterolemia, with accompanying shifts in HDL subclass distributions. These findings are hypothesis-generating and require confirmation in larger, long-term trials.

## 1. Introduction

Beyond raising circulating high-density lipoprotein cholesterol (HDL-C) concentrations, HDL functionality has emerged as a critical determinant of atherosclerotic cardiovascular disease (ASCVD), a major cause of death worldwide [1,2]. HDL exhibits a broad spectrum of anti-atherogenic properties that are not readily reflected by HDL-C levels, including cholesterol efflux capacity (CEC), antioxidant activity against low-density lipoprotein (LDL) oxidation, and anti-inflammatory effects [3,4]. Furthermore, HDL ameliorates endothelial dysfunction, an early hallmark of atherosclerosis characterized by impaired flow-mediated dilation (FMD) of the arterial wall, which is primarily attributed to reduced endothelial nitric oxide synthase (eNOS) activity and the subsequent decline in nitric oxide (NO) bioavailability [5,6]. Accumulating preclinical and clinical evidence demonstrates that HDL enhances eNOS activity promoting ATP-binding cassette transporter G1 (ABCG1)-mediated endothelial 7-ketocholesterol efflux [7,8]. Nevertheless, there are currently no clinical therapeutics capable of improving HDL function to restore endothelial eNOS activity via the ABCG1-dependent 7-ketocholesterol efflux pathway.

Phytochemicals derived from Traditional Chinese Medicine (TCM) hold promising potential for the development of novel anti-atherosclerotic drugs. Protocatechuic acid (PCA), a ubiquitous phytochemical classified within the phenolic acid subclass of polyphenols, is abundant in some TCM (e.g., Chicory, Danshen, and Duzhong) and also widely occurs in plant foods, such as lettuce, Brussels chicory (one cultivated variety of chicory), and olive oil [9,10,11]. This small phenolic compound has a marked atheroprotective property in different mouse models, along with an increase in endothelial function and eNOS activity [12,13]. PCA could directly increase eNOS activity [14]; however, it remains unknown whether this compound could indirectly increase eNOS activity by improving HDL function in facilitating endothelial 7-ketocholesterol efflux. Of note, we previously observed that PCA could improve HDL function in facilitating ABCG1-macrophage cholesterol efflux [15]. Moreover, our recent small-scale randomized controlled trial has further shown that postprandial HDL isolated from overweight individuals after one-week consumption of Brussels chicory rich in PCA increases eNOS activity by promoting ABCG1-mediated endothelial 7-ketocholesterol efflux to HDL [16]. To this end, we hypothesized that individual PCA is a potential boost on eNOS activity by increasing ABCG1-mediated endothelial 7-ketocholesterol efflux to HDL.

## 2. Materials and Methods

### 2.1. Study Design

This 12-week randomized, double-blind, placebo-controlled trial was conducted between November 2022 and May 2023 from physical examination centers in the first, second, third, and sixth affiliated hospitals of Sun Yat-sen University in Guangzhou, Guangdong, China. This trial was conducted in line with the guidelines in the Declaration of Helsinki and approved by the Medical Ethics Committee of the School of Public Health, Sun Yat-sen University, on 1 November 2022 (No. 2022-142). Written informed consent was obtained from each participant before conducting experiments. This trial was registered at the Chinese Clinical Trial Registry (ChiCTR2300067756; www.chictr.org.cn/showprojEN.html?proj=188541, accessed on 15 September 2026) on 20 January 2023.

### 2.2. Randomization and Blinding

Eligible participants were randomly assigned in a 1:1 ratio to receive either PCA or control capsules using a stratified permuted block randomization procedure. Randomization was performed with block sizes of 4 and stratified by sex. Sequentially numbered, opaque, sealed envelopes containing the group allocation were prepared by the same independent statistician and stored in a locked cabinet. Research staff opened the envelopes in strict sequential order only after a participant was confirmed eligible and had provided written informed consent. Participants, investigators, outcome assessors (including sonographers), and statisticians were all blinded to group assignment throughout the trial.

### 2.3. Participants

Participants were included in the present study if they met the following criteria: (1) aged between 35 and 65 years, body mass index (BMI): 24–30 kg/m^2^, permanent resident of Guangzhou City (those who have lived in Guangzhou for more than 10 years); (2) the concentration of total cholesterol in fasting blood between 200–310 mg/dL (5.2–8.0 mmol/L); (3) smoking index less than 400 (smoking index = number of cigarettes smoked per day × smoking years); (4) weekly alcohol consumption less than 175 g of alcohol; (5) the cumulative time of weekly high-intensity exercise not exceeding 75 min or the cumulative time of moderate-intensity exercise not exceeding 150 min; and (6) voluntary participation in the study and signing the informed consent form. The exclusion criteria were as follows: (1) patients who have a serious chronic disease that may affect this trial, such as tumors, chronic heart failure, severe depression or other mental disorders, physical disabilities, or inability to move freely; (2) patients currently taking or having taken any drugs that affect blood lipids and blood sugar in the past six months; (3) patients with poor compliance; and (4) patients who have suffered from acute and chronic infectious diseases, autoimmune diseases, malignant tumors, trauma, or surgery within one month before the test.

### 2.4. Interventions and Follow-Up

PCA and placebo capsules were provided by the Amway Corporation (Ada, MI, USA). Each PCA capsule contains 20 mg PCA, 226.225 mg maltodextrin, and 2.875 mg magnesium stearate (both maltodextrin and magnesium stearate helped to maintain the stability of the PCA), whereas the placebo capsules contained only maltodextrin and magnesium stearate. The aerobic plate count, coliforms, Salmonella, Staphylococcus aureus, molds, and yeasts detected in both PCA capsules and placebo capsules were all below the limits specified in GB 4789.2-2016, GB 4789.3-2016, GB 4789.4-2016, GB 4789.10-2016, and GB 4789.15-2016, respectively [17,18,19,20,21] (Supplementary Information S1 in the Chinese-language version, Supplementary Information S2 in the English-language version). The purity of PCA (CAS 99-50-3, batch number TZ20220703-1) in the PCA capsules was 99.63% according to the manufacturer’s quality report (Supplementary Information S3 in the Chinese-language version, Supplementary Information S4 in the English-language version). PCA and placebo capsules were similar in terms of weight, appearance, and packaging.

One hundred fifty eligible participants were randomly divided into 2 groups to receive either PCA or placebo capsules for 12 weeks and required to consume one capsule daily with dinner during the 12-week intervention period. The participants were followed up through every 6-week face-to-face consultations and weekly phone or WeChat (a widely-used Chinese instant-messaging application) check-ins to evaluate compliance. Throughout the trial, the participants disclosed missed doses, concurrent medication and dietary supplement usage, and adverse events and were instructed to maintain their habitual diet and lifestyle. Dietary intake and the physical activity level over the preceding week were assessed using a three-day 24 h dietary recall and a physical activity questionnaire at the baseline, week 6, and week 12 visits, respectively. At baseline, week 6, and week 12, all participants were required to fast overnight to enable blood sample collection the next morning and to measure FMD and anthropometric characteristics.

### 2.5. Primary Outcome

Brachial artery FMD was assessed as we previously described using a high-resolution vascular ultrasound system (UNEX EF18G; Nagoya, Japan) equipped with a linear-array transducer and automated edge-detection software for continuous measurement of brachial artery diameter [16]. The entire examination procedure was performed in a quiet, temperature-controlled room (20–25 °C) by an experienced ultrasonographer between 8 a.m. and 12 p.m. Prior to testing, participants were instructed to fast for at least 12 h; abstain from alcohol, caffeine, tea, smoking, and polyphenol-rich foods or supplements for at least 12 h; and refrain from strenuous physical exercise for at least 24 h. After 10–15 min of supine rest with the arm positioned at heart level, the brachial artery was imaged longitudinally. An ultrasound transducer was placed on the upper arm, with its lower edge 2–3 cm proximal to the antecubital crease to obtain a longitudinal view of the brachial artery. After baseline brachial-artery diameter recording, the forearm cuff was then inflated to 200 mmHg and maintained for 5 min to induce ischemia. Upon cuff deflation, arterial diameter was continuously tracked until peak vasodilation. Electrocardiogram gating was applied throughout image acquisition. All B-mode sequences and Doppler spectra were digitally recorded and stored as uncompressed video clips (AVI format, frame rate ≥ 30 Hz) on a local server for off-line analysis by a single observer who was blinded to participant characteristics and group allocation. Brachial artery FMD was calculated as the percentage change relative to baseline: [(maximal diameter − baseline diameter)/baseline diameter] × 100%. Intra-observer and inter-observer coefficient of variation (CV) were below 8.9%. Data quality was monitored during acquisition by rejecting scans with an insonation angle > 70°, poor wall visualization for >3 consecutive cardiac cycles, probe displacement > 1 mm, or a baseline diameter CV > 2% within the 60-s recording period.

### 2.6. Secondary Outcomes

Serum PCA was quantified by HPLC-MS as we previously documented [15]. Serum lipids and apolipoproteins [total cholesterol (TC), total triglyceride (TG), LDL cholesterol (LDL-C), HDL-C, apolipoprotein AI (Apo AI)], and glucose were measured using a Cobas e602 automatic biochemical analyzer (Roche Diagnostics, Basel, Switzerland). Blood pressure was measured using a validated blood pressure monitor (Omron, Kyoto, Japan). Participants were seated quietly for at least 5 min in a temperature-controlled room before each measurement, with the right arm supported at heart level and an appropriate-sized cuff placed on the upper arm. Three readings were obtained at intervals of at least 1 min, and the mean of the three readings was calculated. Serum 7-ketocholesterol was detected with commercial kits (No. CB19273-Hu, COIBO BIO, Shanghai, China). Serum cyclic guanosine monophosphate (cGMP) and activities of lecithin-cholesteryl acyltransferase (LCAT), phospholipid transfer protein (PLTP), and cholesteryl ester transfer protein (CETP) activities were measured by corresponding commercially available kits (Merck KGaA, Darmstadt, Germany). Apo AI contents of HDL subclasses were determined by 2-dimensional gel electrophoresis coupled with immunodetection for Apo AI as described previously [22].

HDL functionality was evaluated through in vitro cell assays. Human aortic endothelial cells (HAECs, PCS-100-011, ATCC, Manassas, VA, USA) were first exposed to 7-ketocholesterol (7-KC, 5 µg/mL, 700046P, Merck KGaA, Darmstadt, Germany) for 24 h and then treated with cell culture medium supplemented with 20 µg/mL HDL obtained from study participants for an additional 16 h. eNOS activity, reflected by Ca^2+^-dependent NO synthase enzymatic activity, was quantified via the conversion of [^3^H] L-arginine to [^3^H] L-citrulline using commercially available assay kits (MAK407, Merck KGaA, Darmstadt, Germany). For the assessment of endothelial 7-KC efflux capacity, lipids were extracted from both culture media and cellular fractions, and 7-KC levels were subsequently quantified by gas chromatography as we previously described [16,23]. The 7-KC efflux percentage was computed as the proportion of 7-KC mass present in the medium relative to the total 7-KC mass (medium plus cellular). All experiments were performed in triplicate. The intra- and inter-assay coefficients of variation for eNOS activity and endothelial 7-KC efflux capacity were below 7.7% and 4.7%, respectively. To account for inter-plate variability, a pooled serum standard from all participants at baseline was included on every plate, and all values were normalized against this control pool. HAECs were transfected with 100 nM ABCG1 siRNA (siABCG1) or nontargeting siRNA (siCtr) (Dharmacon, Lafayette, CO, USA), and the knockdown efficiency of ABCG1 was assessed by Western blot and quantitative real-time polymerase chain reaction analysis as we described previously [16]. For HDL function assays, HDL (d = 1.063–1.21 g/mL) was isolated by sequential ultracentrifugation as we previously described [16].

### 2.7. Safety Outcomes

Physical assessments (weight, heart rate, and blood pressure), and blood safety indices [red blood cell, white blood cell, hemoglobin, creatinine, bilirubin, urea, total protein, alanine aminotransferase (ALT), and aspartate transaminase (AST)] were assayed as previously described [24].

### 2.8. Statistical Analysis

The sample size was estimated using PASS software (V11.0, NCSS, LLC, Kaysville, UT, USA.). Sample size was calculated to detect a between-group difference of 1.5% in the mean change in brachial artery FMD between the PCA group and the control group, assuming a standard deviation (SD) of 2.68% [25,26], a power of 90% (β 10%), and an α level of 0.05. This yielded a required sample size of 67 participants per group. Assuming an overall study dropout rate of 10%, we planned to recruit a total of 150 participants for a 2-arm study (*n* = 75 per group).

Descriptive statistics were used to display the baseline demographics, blood index, and daily nutrient intakes of each group. Normal distributions were tested using the Kolmogorov–Smirnov test. Log transformation of the data was performed to achieve normality when required. Variables were presented as mean ± SEM for normal distribution, median, upper and lower quartiles for continuous nonnormally distributed variables, or group proportions for categorical variables. Estimates of outcomes were presented as mean with 95% confidence intervals (CI).

Changes from baseline in brachial artery FMD and other continuous outcomes are reported as raw mean (95% CI). The analysis was performed on the completed population (participants who finished the 12-week intervention). The participants who withdrew from the intervention had no follow-up data and were excluded from outcome analyses. There were no missing outcome data among the analyzed participants at any visit, and no imputation was required or applied. Analysis of linear mixed effects models was conducted to assess the impact of PCA supplementation on clinical outcomes by restricted maximum likelihood. The models included time, PCA intervention, and the interaction of the PCA intervention by time to allow for differing effects of the intervention by time. An unstructured residual covariance matrix across visits was specified to account for the correlation between repeated measurements, and no random effects were fitted. When comparing the changes of the outcomes, age, sex, and baseline value of each outcome were included as covariates. Correlations between changes in eNOS activity of participants and changes in the abundance of HDL subgroups, brachial artery FMD, cGMP, and endothelial 7-ketocholesterol efflux capacity were calculated using Spearman test. The primary outcome was brachial artery FMD, and all other outcomes, including secondary outcomes, subgroup analyses, and correlation analyses, were pre-specified as exploratory. No adjustment for multiple comparisons was applied to these exploratory analyses. Data were analyzed using Stata version 16.0 (StataCorp LP, College Station, TX, USA) and GraphPad Prism version 8.4.0.

## 3. Results

### 3.1. Participants and Compliance

**Figure 1:**
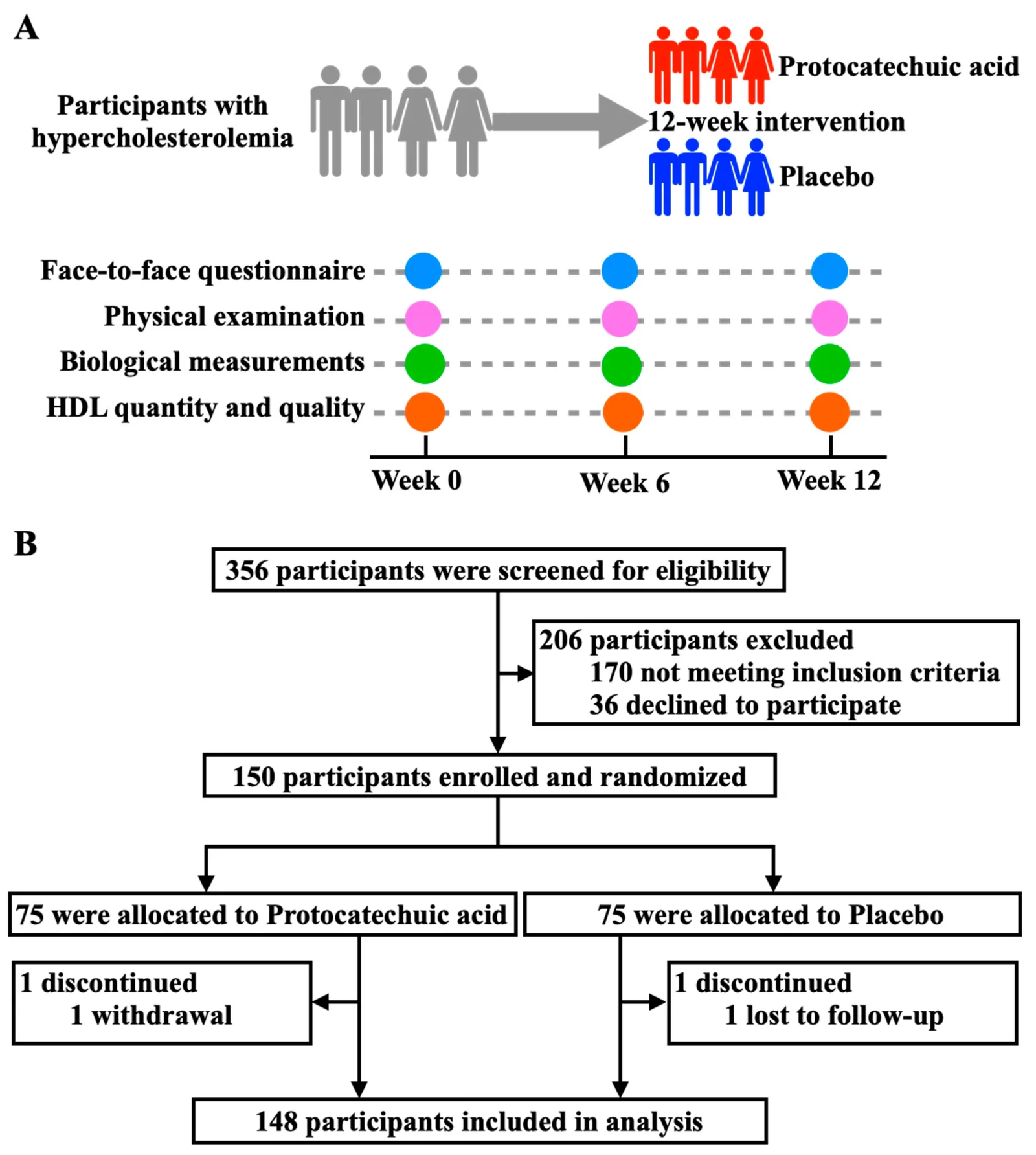
A summary of the study design. (**A**) The schematic diagram of the study design. (**B**) The flowchart of enrollment and randomization.

As a result of the screening, we selected 150 participants eligible to participate in the clinical study (Figure 1). Two participants (one per group) withdrew within the first 10 days of the intervention for personal reasons and were excluded from the analyses of outcome changes. Table 1 summarizes the anthropometric characteristics of all 150 randomized participants. There were no significant differences in baseline characteristic and daily mean energy and nutrient intakes between the two studied groups (Table 1, Supplementary Table S1). No participants reported any adverse events resulting from the consumption of either the placebo or PCA capsules during the trial period. Among the analyzed participants, 148 (74 per group) had available outcome data at both week 6 and week 12, with no missing observations at any visit.

According to the count of the recalled capsules at every visit, compliance was quite good. The rates of capsule intake were 94.1% and 94.8% in the placebo and PCA groups, respectively. Compliance in the PCA group was also confirmed by the increased levels of serum PCA after the 6- or 12-week of PCA intake, whereas the levels of serum PCA in the placebo group were similar during the trial period (Table 2).

### 3.2. Primary Outcome

There were increases in the brachial artery FMD (95% CI) after a 12-week PCA intervention [adjusted difference from control, 1.77% (95% CI: 1.46 to 2.08); *p* < 0.001]. Mirroring the increased brachial artery FMD, the serum cGMP concentrations also increased after a 12-week PCA intervention [difference, 11.72 pmol/L (95% CI: 6.16 to 17.28); *p* < 0.001] (Table 2, Supplementary Figure S1). Similar patterns in men (*n* = 81), women (*n* = 67), younger (35–49 y; *n* = 103), and older (50–65 y; *n* = 45) participants in the response to the PCA or placebo capsules intervention were observed (Supplementary Figure S2).

### 3.3. Secondary Outcomes

Serum concentrations of serum lipids, apolipoproteins, 7-kcl, glucose, insulin and HOMA-IR, diastolic blood pressure, and heart rate did not differ between the placebo and PCA groups at baseline and after the 12-week intervention (Supplementary Table S2). In addition, no differences in side effects indicated by non-significant changes in hematological indices and liver and kidney function among the two groups were observed (Supplementary Table S3).

Of note, there were significant increases in the HDL_3c_, HDL_3b_, and HDL_3a_ after a 12-week PCA intervention [difference, 19.97 mg/L (95% CI: 12.54 to 27.39), 34.21 mg/L (95% CI: 24.36 to 44.06), 15.55 mg/L (95% CI: 4.84 to 26.25), respectively] (Table 2). In contrast, the serum HDL_2a_ and HDL_2b_ concentrations had been reduced after a 12-week PCA intervention [difference, −43.57 mg/L (95% CI: −56.58 to −30.56), −47.52 mg/L (95% CI: −61.85 to −33.20), respectively]. There were no significant changes in serum pre-β_1_-HDL and pre-β_2_-HDL at 12 weeks in the PCA vs. placebo control. Similar patterns in men, women, younger (35–49 y), and older (50–65 y) participants in the response to the PCA or placebo control were observed (Supplementary Figure S2). There were no significant differences in the enzymatic activities of serum LCAT, PLTP, and CETP, three well-known enzymes involved in regulating HDL subclass distribution, between the placebo and PCA groups at the 12-week intervention.

**Figure 2:**
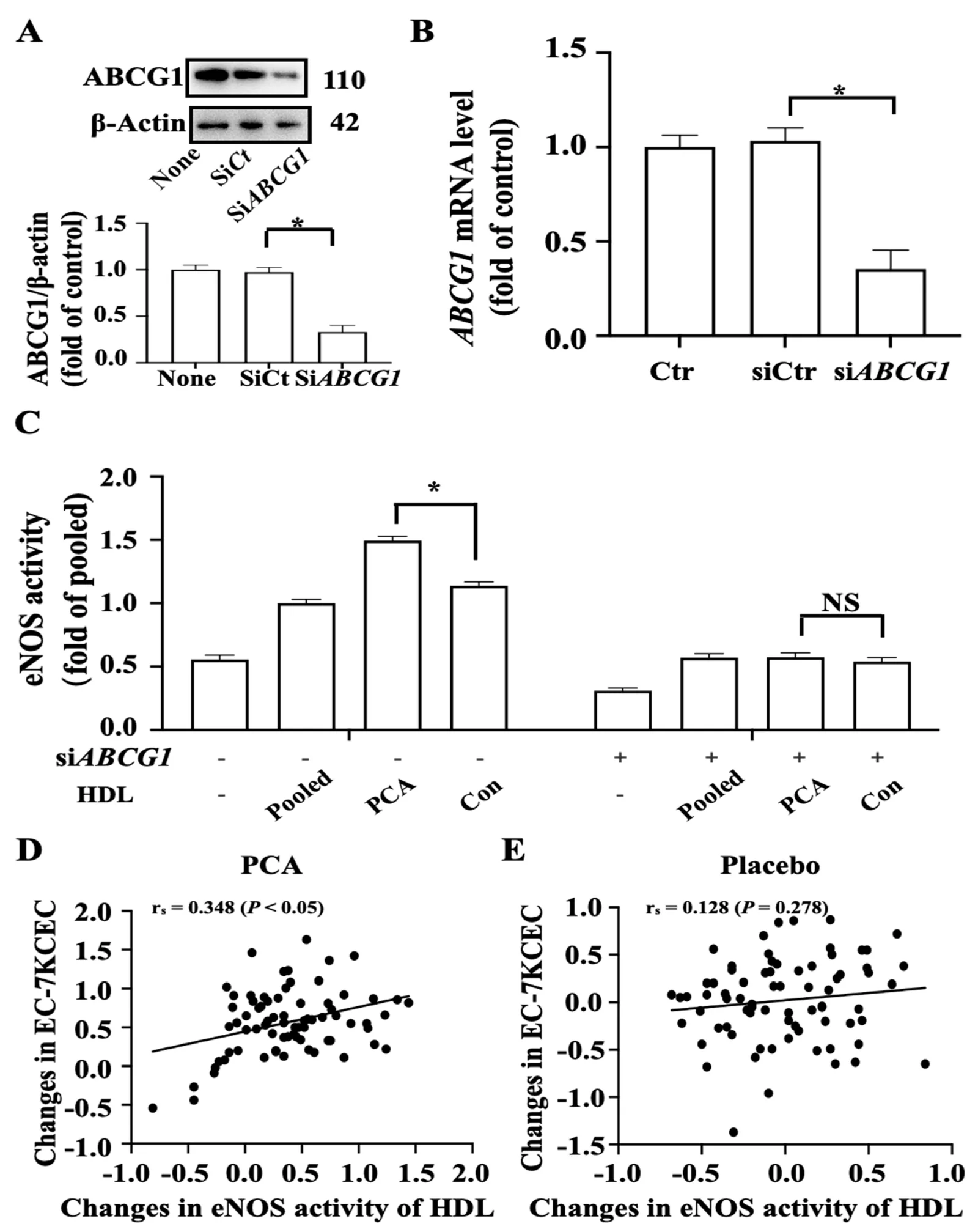
HDL-mediated eNOS activation was functionally reliant on EC-7KCEC. (**A**,**B**) HAECs were untreated or transfected with control siRNA (siCtr) or siRNA directed against ABCG1 (siABCG1) with 100 nM for 24 h. ABCG1 knockdown efficiency was then determined by Western blot (**A**) and qRT-PCR (**B**) assays. (**C**) 7-KC-loaded HAECs that had been transfected with ABCG1 siRNA or control siRNA were incubated with HDL isolated from participants after dietary intervention for 16 h. eNOS activities in HAECs was then measured. The pooled serum from all subjects at the baseline was served as the control group. (**D**,**E**) Correlation between the changes in eNOS activity of HDL and the changes in EC-7KCEC of HDL in PCA (**D**) and Con (**E**). Data were mean ± SEM (*n* = 6, **A**,**B**; *n* = 148, **C**–**E**). * *p* < 0.05, NS indicates not significant. 7-KC, 7-ketocholesterol; ABCG1, ATP-binding cassette transporter G1; Con, control; HAECs, human aortic endothelial cells; EC-7KCEC, cultured human aortic endothelial 7-ketocholesterol efflux capacity; eNOS, endothelial nitric oxide synthase; HDL, high-density lipoprotein.

In parallel with the changes in HDL subclass distribution, there were significant increases in HDL-induced eNOS activation [difference, 0.39 (95% CI: 0.29 to 0.49)] and in endothelial 7-ketocholesterol efflux capacity [difference, 0.44 (95% CI: 0.34 to 0.53)] in 7-ketocholesterol-laden HAECs after a 12-week PCA intervention (Table 2). Consistently, PCA intervention significantly reduced systolic blood pressure as compared with the placebo treatment [difference, −8.24 (95% CI: −10.22 to −6.26)] (Supplementary Table S2). Given that HDL could enhance eNOS activity via facilitating ABCG1-mediated endothelial 7-ketocholesterol efflux, we employed siRNA technology to silence the expression of endothelial ABCG1. The knockdown efficiency exceeded 74% in HAECs, as confirmed by substantial reductions in both mRNA and protein levels (Figure 2A,B). As illustrated in Figure 2C, ABCG1 silencing abrogated the stimulatory effects of HDL isolated from participants following PCA intervention on eNOS activity in 7-ketocholesterol-laden HAECs. Correlation analyses further demonstrated that alterations in HDL-mediated eNOS activity exhibited a significant positive association with corresponding changes in endothelial 7-ketocholesterol efflux capacity in PCA group (Figure 2D). In contrast, no such relationship was observed between these variables in the control group (Figure 2E).

### 3.4. Correlation Between Changes in HDL Functionality and HDL Subclass Distribution

**Figure 3:**
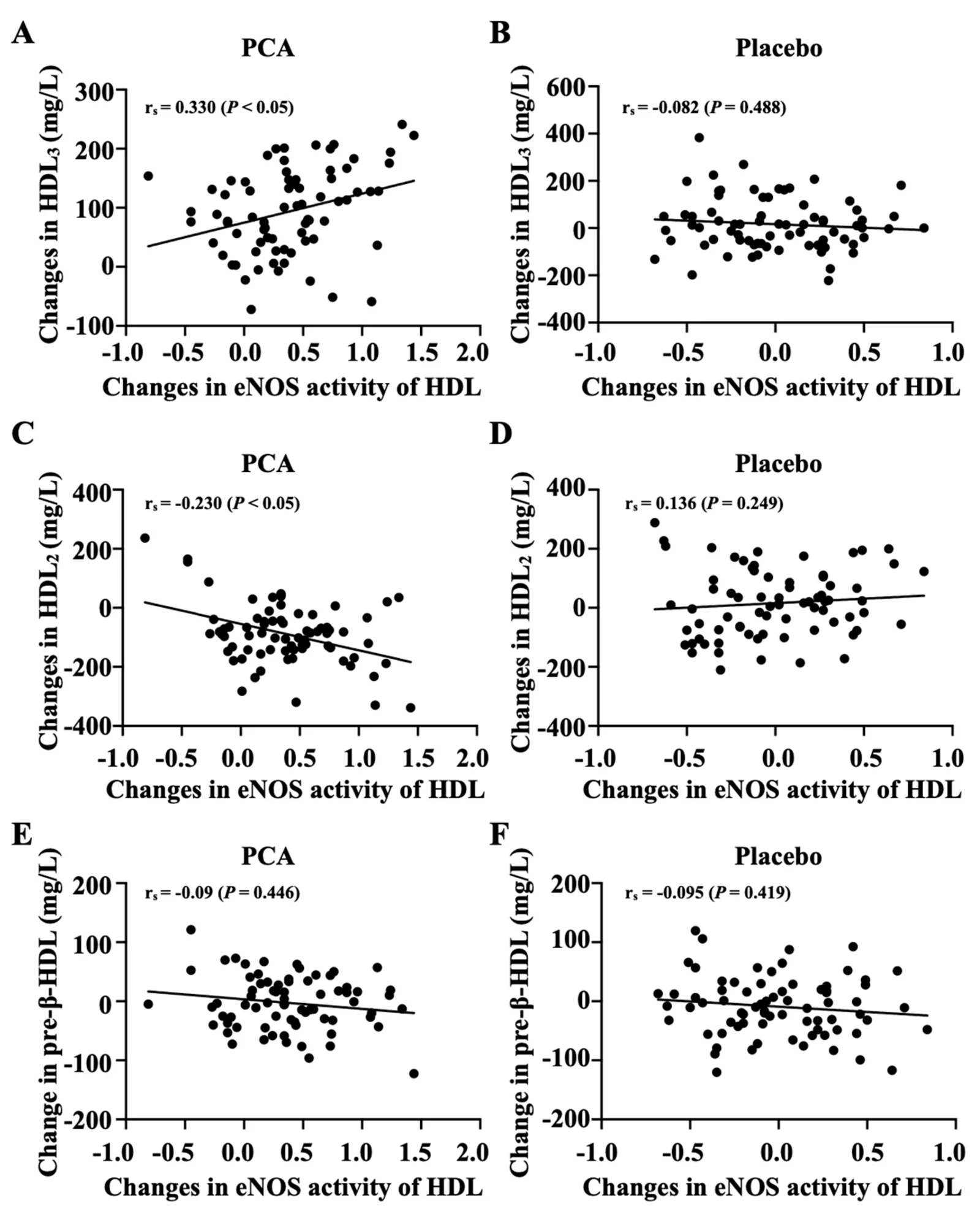
The correlation between the changes in eNOS activity of HDL and the changes in HDL subclass distribution. The correlation between the changes in eNOS activity of HDL and the changes in the variation of HDL_3_ (**A**,**B**); the variation in HDL_2_ (**C**,**D**) and the variation in pre-β-HDL (**E**,**F**) are shown. Spearman correlation coefficients are noted for each plot. HDL, high-density lipoprotein; eNOS, endothelial nitric oxide synthase; PCA, protocatechuic acid.

The changes in HDL-induced eNOS activation were positively correlated with the changes in HDL_3_ content in the PCA group (Figure 3A), whereas no correlation was observed in the placebo group (Figure 3B). This change also showed a negative correlation with the change in HDL_2_ content in the PCA group (Figure 3C) but not in the placebo group (Figure 3D). Moreover, there was no significant correlation between the changes in HDL-induced eNOS activation and the changes in pre-β-HDL in each study group (Figure 3E,F).

### 3.5. Correlation Between Changes in HDL Functionality and Those in Brachial Artery FMD or Serum cGMP Content

**Figure 4:**
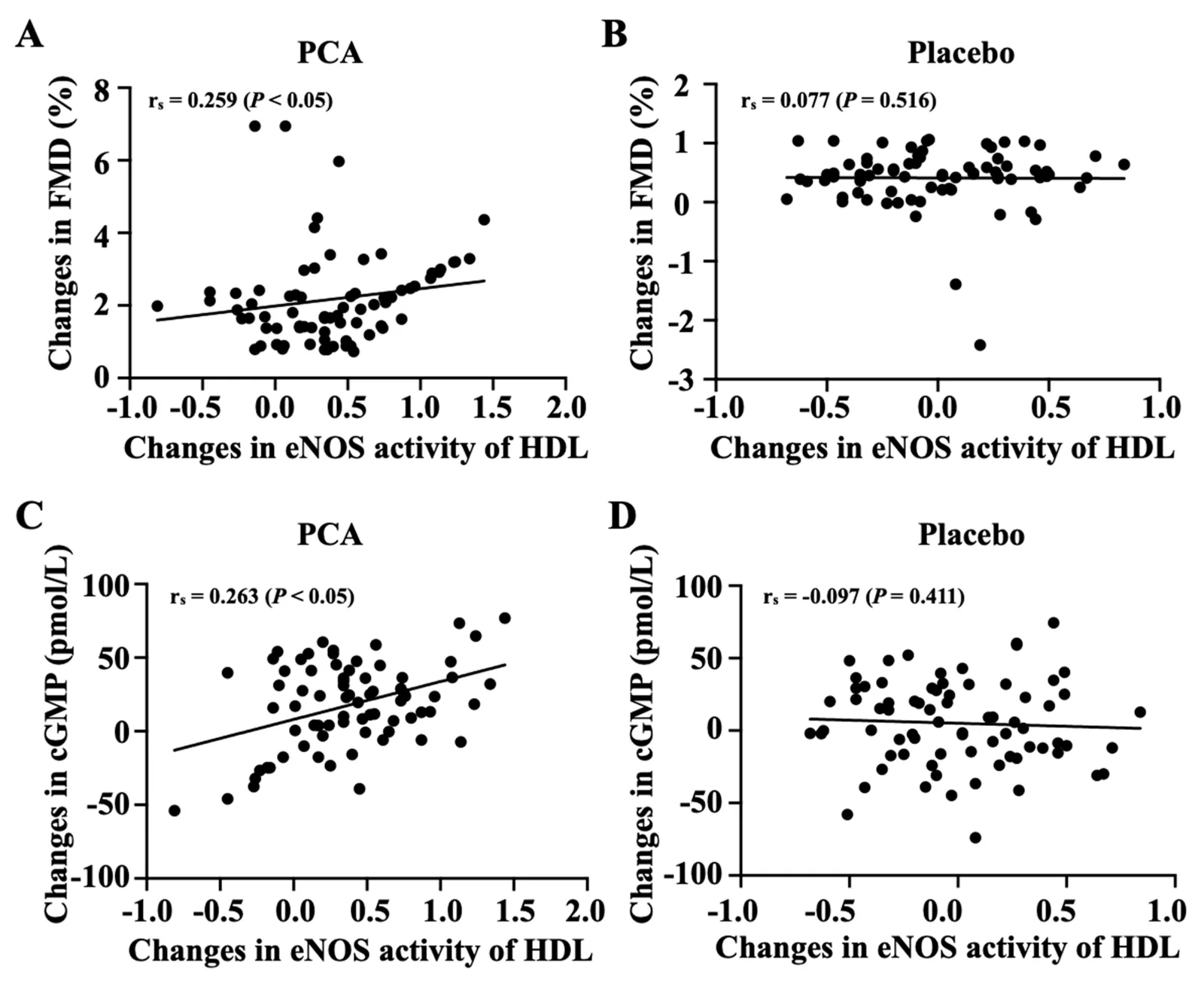
The correlation between the changes in eNOS activity of HDL and the changes in brachial artery FMD and cGMP. The correlation between the changes in eNOS activity of HDL and the changes in brachial artery FMD (**A**,**B**) and cGMP (**C**,**D**) are shown. Spearman correlation coefficients are noted for each plot. cGMP, cyclic guanosine monophosphate; eNOS, endothelial nitric oxide synthase; FMD, flow-mediated dilation; PCA, protocatechuic acid.

In the PCA group, the changes in HDL-induced eNOS activation were positively correlated with the changes in brachial artery FMD; however, no correlation between these changes in the placebo group was observed (Figure 4A,B). Similar to the relationship between HDL-induced eNOS activation and brachial artery FMD, there was a positive correlation between the changes in HDL-induced eNOS activation and the changes in serum cGMP content in the PCA group but not in the placebo group (Figure 4C,D).

## 4. Discussion

Preclinical studies have strongly suggested that PCA is able to reduce the risk or severity of ASCVD [10,27,28]. However, to our knowledge, there are no clinical trials that aimed at translating the health-promoting effect of PCA. Herein, using a randomized double-blind placebo-controlled trial in adult participants with hypercholesterolemia, we observed three major findings: (i) Dietary supplementation of PCA for 12 weeks promoted brachial artery FMD. (ii) PCA consumption increased HDL atheroprotective function characterized by increased activities of eNOS, a key enzyme involving in the process of FMD; (iii) the increased HDL atheroprotective function was associated with more smaller HDL particles (HDL_3_) and less larger HDL (HDL_2_) particles, which likely resulted in an increase in HDL function in ABCG1-mediated 7-ketocholesterol efflux from endothelial cells. These novel findings thus allow us to propose that dietary supplementation of PCA could promote FMD, possibly through an improvement of HDL function on eNOS activity through augmented HDL function in ABCG1-mediated 7-ketocholesterol efflux capacity by shifting its particle size toward smaller values. Nevertheless, further clinical trials with a long-term intervention are required to uncover whether the benefits of PCA on FMD and also HDL subclass distribution and functionality could prevent acute ASCVD events, such as myocardial infarction and stroke, the leading causes of mortality worldwide.

The benefits of PCA on FMD in adult participants with hypercholesterolemia may have an important implication. The dose of PCA required to promote FMD (20 mg) is clinically meaningful, as this dose is readily achieved through a regular diet. An amount of 100 g of lettuce or French endive (a typical leafy vegetable in Mediterranean countries) often have an average of 20 mg PCA [29,30]. These observations thus allow us to propose that PCA is a promising candidate to prevent ASCVD by consumption of purified PCA or PCA-rich foods.

eNOS plays a critical role in preserving endothelial function and promoting FMD via the conversion of L-arginine into nitric oxide [31,32]. eNOS activity is finely regulated by multiple factors, one of which is the interaction of arterial endothelial ABCG1 with circulating HDL. This interaction promotes the efflux of 7-ketocholesterol, an oxidized form of cholesterol that is often accumulated in arterial endothelial cells in the context of atherosclerosis and subsequently increases eNOS activity [16]. Herein, we observed that HDL isolated from adult participants with hypercholesterolemia after PCA consumption has a higher ability to increase eNOS activities by facilitating endothelial ABCG1-mediated 7-ketocholesterol efflux. This finding thus suggests that the improved HDL functionality on eNOS activities is one potential mechanism underlying the PCA effect on FMD in adult participants with hypercholesterolemia. However, we did not test the possibility of whether PCA itself or its glucuronide- and sulfate-conjugated metabolites could directly increase eNOS activity and FMD in adult participants with hypercholesterolemia. Indeed, Chen’s group has recently demonstrated that PCA at 10–100 nmol/L increases eNOS activities in IL-1β-stimulated endothelial cells [14]. These doses are comparable with these concentrations in blood circulation after PCA consumption in adult participants with hypercholesterolemia.

HDLs are a class of structurally and functionally heterogeneous particles. Using 2-dimensional polyacrylamide gel electrophoresis and subsequent immunoblotting method, HDL particles can be divided into large spherical subclasses (HDL_2a_ and HDL_2b_), intermediate spherical subclasses (HDL_3c_, HDL_3b_ and HDL_3a_), small discoidal subclass pre-β_1_-HDL, and large discoidal subclass pre-β_2_-HDL. Numerous studies have investigated the varied role of different HDL subclasses in macrophage cholesterol efflux capacity [33], perhaps the most studied atheroprotective functionality of HDL, but these impacts on eNOS activity, one of atheroprotective functions of HDL, remains to be established. Our current studies have shown that in the PCA group, increased HDL-induced eNOS activation capacity were negatively and positively correlated with a reduction in HDL_2_ content and an increase in HDL_3_ content, respectively. These findings are compatible with the hypothesis that intermediate spherical subclasses of HDL (HDL_3_) might be better than large spherical subclasses (HDL_2_) in terms of increasing eNOS activity. It should be borne in mind that the observed correlations between HDL subclass profiles and HDL functionality do not establish a causal relationship whereby HDL subclass remodeling drives endothelial effects. Nevertheless, our current findings should be regarded as hypothesis-generating for future mechanistic investigations. To directly validate this hypothesis, further studies assessing the effects of isolated HDL_3_ and HDL_2_ fractions on eNOS activity both in vitro and in vivo are warranted.

It should be pointed out that PCA-rich Brussels chicory consumption did not appreciably affect brachial artery FMD [16], though PCA-rich Brussels chicory and individual PCA have a similar potency to increase HDL function in eNOS activity and shift HDL particle sizes toward smaller values. The exact reason for this discrepancy is presently unclear. It is probable that some unidentified components in Brussels chicory could impair brachial artery FMD, thus covering the PCA effect. On the other hand, different participants (hypercholesterolemia in the PCA trial versus overweight without hypercholesterolemia in the Brussels chicory trial) and different intervention periods (12 weeks in the PCA trial versus 1 week in the Brussels chicory trial) may also be responsible for these inconsistencies. Indeed, hypercholesterolemia is often associated with impaired FMD that is partly due to exposure to higher level of circulating 7-ketocholesterol [34]. Supporting this speculation, the concentration of circulating 7-ketocholesterol in participants with hypercholesterolemia from the PCA trial was almost two-fold higher than that in participants with overweight from the Brussels chicory trial. These observations suggest that the effect of PCA-rich Brussels chicory or individual PCA on FMD may be modulated by different health states.

In addition to being a natural phenolic acid, PCA is also one gut microbiota-derived metabolite of anthocyanins [15,27]. Interventional studies in humans and experimental animals from our and other groups have consistently reported that dietary supplementation of anthocyanins or anthocyanin-rich foods (e.g., blueberry and Acai berry) is able to reduce the risk or severity of ASCVD, including promoting of FMD [35,36,37]. Consistently, cohort studies have also shown that high anthocyanin intake is associated with a reduced risk of myocardial infarction in young and middle-aged women [38]. Of note, mechanistic studies further uncovered that PCA is largely responsible for the atheroprotective effect of its precursor the anthocyanins in ApoE^−/−^ mice [15]. Together with our current findings that dietary supplementation of PCA for 12 weeks promoted FMD in adult participants with hypercholesterolemia, we hypothesize that the effect of anthocyanins on FMD observed in humans may be partially linked to its gut microbiota metabolite PCA. This hypothesis is worthy to be tested, as it would further broaden the understanding of the mechanisms underlying the benefits of anthocyanins on the cardiovascular system.

Of note, compared with the placebo group, PCA supplementation lowered SBP by approximately 8 mmHg at week 12, with no corresponding change in DBP. This divergent response has also been reported in several previous polyphenol-related randomized trials and meta-analyses, in which dietary polyphenol interventions exerted favorable effects primarily on SBP rather than DBP [39,40]. Systolic blood pressure is more sensitive to alterations in large-artery elasticity and NO-dependent endothelial vasodilation, whereas DBP is jointly modulated by peripheral vascular resistance, heart rate, and vascular volume [41], which may partly explain this discrepancy. Nevertheless, since SBP and DBP were secondary outcomes in our trial, this finding should be interpreted cautiously and requires confirmation in larger-scale dedicated trials.

The current study has several limitations. First, although we observed that PCA-elicited improvement in FMD was closely associated with increased functionality of HDL on eNOS activity, we could not quantify its exact contribution to the improved FMD. Second, although PCA consumption did not affect the activities of LCAT, PLTP, and CETP, three well-known enzymes to modulate HDL subclass distribution [42], the exact mechanisms underlying the PCA effect on HDL subclass distributions still remain elusive. Third, the intervention was conducted in adult participants with hypercholesterolemia. However, the results need to be carefully extended to the other pathological and/or physiological populations. Additional randomized controlled trials are still needed to assess the potential benefits of PCA consumption in individuals under different health conditions. Fourth, as we did not collect baseline data on habitual dietary polyphenol (e.g., PCA, anthocyanin) intake, background dietary polyphenol consumption may represent a potential confounder for the present findings. Fifth, the secondary outcomes, subgroup analyses, and correlation analyses were exploratory and not adjusted for multiple testing. Accordingly, these findings may be subject to type I error and should be regarded as hypothesis-generating rather than confirmatory. Last but not least, the trial was registered after enrolment had commenced (first participant enrolled in November 2022; registered on 20 January 2023) owing to administrative processing delays. Although this represents a deviation from prospective registration, the study protocol and statistical analysis plan were finalized and locked prior to database unblinding, ensuring that all pre-specified analyses remained unbiased.

## 5. Conclusions

Taken together, our clinical trial results have shown for the first time that PCA supplementation in adults with hypercholesterolemia is associated with increased FMD, a shift in HDL particles toward small sizes, and improved HDL functionality (enhanced eNOS activity and ABCG1-mediated endothelial 7-ketocholesterol efflux). Nevertheless, these observations remain hypothesis-generating and require confirmation in larger, long-term trials.

## Figures and Tables

**Table 1:** The baseline clinical characterization of the study population ^1^.

Characteristics	Placebo (*n* = 75)	PCA (*n* = 75)
Anthropometric characteristics		
Age, y	45 (42, 50) ^2^	47 (42, 51)
Sex		
Male, *n*	43	39
Female, *n*	32	36
Body weight, kg	75.69 ± 1.30	75.83 ± 1.42
BMI, kg/m^2^	26.91 ± 0.18	27.06 ± 0.15
Waist circumference, cm	85.22 ± 0.57	83.83 ± 0.52
Hip circumference, cm	96.00 ± 0.60	96.66 ± 0.54
Body fat, %	27.79 (25.93, 28.78)	28.46 (26.98, 30.53)
Lifestyle factors		
Smoking, %	48.00	42.67
Physical activity level, MET-min/week	1260.16 (1101.57, 1471.60)	1196.78 (1021.55, 1441.45)
Blood indicators		
TC, mmol/L	6.52 ± 0.07	6.54 ± 0.08
TG, mmol/L	1.42 ± 0.03	1.34 ± 0.03
HDL-C, mmol/L	1.30 ± 0.06	1.41 ± 0.06
Pre-β_1_-HDL, mg/L	94.41 ± 3.37	90.25 ± 2.48
Pre-β_2_-HDL, mg/L	50.24 ± 2.20	48.89 ± 2.35
HDL_3c_, mg/L	73.37 ± 3.06	70.63 ± 2.79
HDL_3b_, mg/L	167.05 ± 3.45	170.90 ± 3.74
HDL_3a_, mg/L	278.15 ± 5.35	277.83 ± 6.02
HDL_2a_, mg/L	267.92 ± 5.53	271.12 ± 4.87
HDL_2b_, mg/L	321.72 ± 6.30	317.19 ± 4.18
LDL-C, mmol/L	2.43 ± 0.04	2.45 ± 0.04
Apo AI, g/L	1.33 ± 0.05	1.28 ± 0.05
Apo B, g/L	1.11 ± 0.05	1.14 ± 0.04
7-KC, nmol/L	38.80 ± 0.53	40.17 ± 0.45
Glucose, mmol/L	4.82 ± 0.05	4.74 ± 0.05
Insulin, mU/L	12.47 (10.30, 14.87)	11.15 (8.70, 14.69)
HOMA-IR	2.67 ± 0.08	2.46 ± 0.09
Red blood cell, ×10^12^/L	4.35 ± 0.04	4.38 ± 0.05
White blood cell, ×10^9^/L	5.36 ± 0.10	5.47 ± 0.11
Hemoglobin, g/L	124.92 ± 1.10	123.07 ± 1.00
Creatinine, μmol/L	93.17 (87.68, 99.87)	94.37 (86.73, 99.04)
Bilirubin, μmol/L	12.07 ± 0.33	11.96 ± 0.35
Urea, mmol/L	5.18 ± 0.22	5.02 ± 0.22
Total protein, g/L	74.79 ± 0.51	74.59 ± 0.47
Albumin, g/L	44.12 ± 0.41	43.94 ± 0.40
AST, U/L	30.03 (25.01, 34.21)	28.85 (23.99, 35.00)
ALT, U/L	30.68 ± 1.07	29.46 ± 1.10
Blood pressure		
SBP, mm Hg	129.27 ± 0.99	130.20 ± 0.80
DBP, mm Hg	74.97 ± 0.93	74.99 ± 1.24
Heart rate, beats/min	73.01 ± 0.85	74.41 ± 0.84

^1^ Values are means ± SEM unless otherwise indicated. ^2^ Median (25th–75th percentile). 7-KC, 7-ketocholesterol; ALT, alanine aminotransferase; Apo AI, apolipoprotein AI; Apo B, apolipoprotein B; AST, aspartate transaminase; BMI, body mass index; DBP, diastolic blood pressure; HDL-C, high-density lipoprotein cholesterol; HOMA-IR, homeostasis model assessment of insulin resistance; LDL-C, low-density lipoprotein cholesterol; PCA, protocatechuic acid; SBP, systolic blood pressure; TC, total cholesterol; TG, triglyceride.

**Table 2:** Changes in FMD, HDL subclass distribution and functionality of study population ^1^.

	6-Week Change ^2^	12-Week Change ^2^	Adjusted PCA-Placebo Difference at Week 12	*p* Value (12 Week) ^3^
PCA, nmol/L				
Placebo	0.43 (0.15 to 0.70)	0.44 (0.15 to 0.72)		
PCA	8.85 (7.55 to 10.14)	16.76 (15.59 to 17.92)	16.36 (15.18 to 17.55)	<0.001
FMD, %				
Placebo	−0.22 (−0.32 to −0.12)	0.41 (0.29 to 0.53)		
PCA	0.67 (−0.15 to 1.48)	2.17 (1.87 to 2.46)	1.77 (1.46 to 2.08)	<0.001
cGMP, pmol/L				
Placebo	3.27 (−4.49 to 11.03)	5.11 (−1.71 to 11.92)		
PCA	7.58 (0.36 to 14.81)	17.89 (11.17 to 24.62)	11.72 (6.16 to 17.28)	<0.001
HDL subclass				
Pre-β_1_-HDL, mg/L				
Placebo	2.45 (−8.07 to 12.96)	2.40 (−8.41 to 13.20)		
PCA	1.36 (−6.64 to 9.37)	4.01 (−4.22 to 12.24)	−4.22 (−10.95 to 2.50)	0.218
Pre-β_2_-HDL, mg/L				
Placebo	5.23 (−1.09 to 11.54)	2.14 (−3.86 to 8.13)		
PCA	2.78 (−3.17 to 8.73)	−0.80 (−6.56 to 4.96)	−3.98 (−8.58 to 0.62)	0.090
HDL_3c_, mg/L				
Placebo	−3.62 (−10.24 to 3.01)	8.08 (−0.74 to 16.90)		
PCA	5.31 (−2.28 to 12.90)	30.89 (23.51 to 38.26)	19.97 (12.54 to 27.39)	<0.001
HDL_3b_, mg/L				
Placebo	6.50 (−3.64 to 16.64)	5.64 (−4.16 to 15.44)		
PCA	3.03 (−6.83 to 12.89)	36.57 (26.35 to 46.79)	34.21 (24.36 to 44.06)	<0.001
HDL_3a_, mg/L				
Placebo	6.08 (−10.61 to 22.77)	3.26 (−10.01 to 16.54)		
PCA	11.71 (−4.24 to 27.67)	29.47 (14.15 to 44.79)	15.55 (4.84 to 26.25)	0.004
HDL_2a_, mg/L				
Placebo	2.33 (−12.33 to 16.98)	18.11 (5.22 to 31.00)		
PCA	−10.71 (−24.52 to 3.09)	−28.79 (−41.62 to −15.95)	−43.57 (−56.58 to −30.56)	<0.001
HDL_2b_, mg/L				
Placebo	12.38 (−4.70 to 29.46)	−12.33 (−28.92 to 4.27)		
PCA	−10.38 (−25.41 to 4.65)	−54.92 (−66.58 to −43.27)	−47.52 (−61.85 to −33.20)	<0.001
HDL metabolism				
LCAT, pmol/μL/h				
Placebo	−0.38 (−2.78 to 2.02)	0.42 (−1.87 to 2.70)		
PCA	−0.38 (−2.86 to 2.09)	−0.84 (−3.25 to 1.57)	0.11 (−1.71 to 1.92)	0.910
CETP, pmol/μL/h				
Placebo	1.29 (−4.64 to 7.22)	4.85 (−1.19 to 10.89)		
PCA	−2.56 (−9.01 to 3.89)	0.57 (−6.32 to 7.45)	−1.17 (−5.34 to 3.00)	0.582
PLTP, pmol/μL/h				
Placebo	−0.29 (−2.52 to 1.94)	0.67 (−1.77 to 3.11)		
PCA	0.23 (−2.27 to 2.74)	0.12 (−2.67 to 2.92)	−0.47 (−2.23 to 1.29)	0.600
HDL function				
eNOS activity ^4^				
Placebo	−0.02 (−0.11 to 0.06)	−0.00 (−0.09 to 0.08)		
PCA	0.10 (−0.01 to 0.20)	0.38 (0.28 to 0.49)	0.39 (0.29 to 0.49)	<0.001
EC-7KCEC ^4^				
Placebo	−0.02 (−0.12 to 0.09)	0.01 (−0.10 to 0.11)		
PCA	0.11 (0.00 to 0.22)	0.46 (0.35 to 0.56)	0.44 (0.34 to 0.53)	<0.001

^1^ All values are means (95% CI). ^2^ Data are presented as raw mean (95% CI) for changes from baseline. ^3^ The main effect of intervention was analyzed by a linear mixed model of repeated measures with terms for treatment, time, and time × treatment interactions as fixed effects with an unstructured residual covariance matrix across visits and no random effects and baseline values, age, and sex as covariates, *p* value for the week-12 contrast. ^4^ eNOS activity and EC-7KCEC were presented as relative levels versus the control pool. *p* < 0.05 indicates a significant difference. CETP, cholesteryl ester transfer protein; cGMP, cyclic guanosine monophosphate; CI, confidence interval; EC-7KCEC, cultured human aortic endothelial 7-ketocholesterol efflux capacity; eNOS, endothelial nitric oxide synthase; FMD, flow-mediated dilation; LCAT, lecithin-cholesteryl acyltransferase; PCA, protocatechuic acid; PLTP, phospholipid transfer protein.

## Data Availability

All data that support the findings of this study are available from the corresponding author upon reasonable request due to privacy and ethical restrictions related to participant confidentiality.

## Supplementary Materials

The following supporting information can be downloaded at https://www.mdpi.com/article/10.3390/nu18183056/s1, Figure S1: Beneficial effect of PCA on endothelial function in participants with hypercholesterolemia; Figure S2: Changes in the outcomes of endothelial function and HDL subclass distribution after a 12-week intervention between PCA and placebo; Table S1: Mean daily intake of nutrients of study population; Table S2: Changes in cardio-metabolic biomarkers of study population; Table S3: Changes in hematological indices, liver and kidney function of study population; Supplementary Information S1. PCA Safety; Supplementary Information S2. Translated version of PCA Safety; Supplementary Information S3. PCA; Supplementary Information S4. Translated version of PCA; Supplementary Information S5. CONSORT checklist. Reference [43] is cited in the supplementary materials.

## Abbreviations

The following abbreviations are used in this manuscript:

7-KC	7-ketocholesterol
ABCG1	ATP-binding cassette transporter G1
ALT	alanine aminotransferase
Apo AI	apolipoprotein AI
ASCVD	atherosclerotic cardiovascular disease
AST	aspartate transaminase
CETP	cholesteryl ester transfer protein
cGMP	cyclic guanosine monophosphate
DBP	diastolic blood pressure
EC-7KCEC	cultured human aortic endothelial 7-ketocholesterol efflux capacity
eNOS	endothelial nitric oxide synthase
FMD	flow-mediated dilation
HAECs	human aortic endothelial cells
HDL	high-density lipoprotein
HDL-C	high-density lipoprotein cholesterol
HOMA-IR	homoeostasis model assessment of insulin resistance
LCAT	lecithin-cholesteryl acyltransferase
LDL-C	low-density lipoprotein cholesterol
NO	nitric oxide
PCA	protocatechuic acid
PLTP	phospholipid transfer protein
SBP	systolic blood pressure
TC	total cholesterol
TCM	Traditional Chinese Medicine
TG	total triglyceride
