# Supplementary material for: Effects of Protocatechuic Acid on Endothelial Function and HDL Functionality in Hypercholesterolemic Subjects: A Randomized Double-Blind Placebo-Controlled Trial
Source: Nutrients. 2026 Sep 18;18(18):3056. doi: 10.3390/nu18183056 (PMC13610876; doi:10.3390/nu18183056)
Supplement: Supplementary file 1 [file nutrients-18-03056-s001.zip › Supplementary Information S1. PCA Safety.pdf]

**检测报告** （报告编号#WA2022025051） 发布日期:2022年08月30日

客户名称： 安利（中国）研发中心有限公司

客户地址： 广州经济技术开发区北围工业区一区临江路 1 号

样品名称： / PCA

生产商/供应商： ACCL

样品批号： （见下页）

生产日期： /

**以上样品及信息由客户提供及确认，AGTC不承担证实客户提供信息的准确性，适当性和（或）完整性责任。**

委托单号： WA2022025051

样品接收日期： 2022年08月23日

样品状态： 包装完好。

检测周期： 2022年08月23日-2022年08月29日

检测要求： 根据客户提供的10134的第5版文件检测。

检测方法： （见下页）

检测结果： （见下页）

**除非另有说明,本检测结果仅与被检测物品有关。仅供客户内部使用,不对社会具有证明作用。未经检验机构书面同意,委托人不得擅自使用检测结果进行不当宣传。**

AGTC 授权签字人

捷通（广州）检测技术服务有限公司
（检验检测专用章）

**声明：**1）捷通（广州）检测技术服务有限公司，简称 ：AGTC。 2）报告未加盖本公司检验检测专用章无效，无相关授权人签字无效。 3）未经本公司书面批准不得部分复制报告，全部复制除外。 4）报告涂改增删无效。5）对送检样品，报告仅对收到的样品负责。6）未经本公司允许，任何人不得使用本报告进行不当宣传。7）对报告的异议应于报告签发之日起15日内向本公司提出，逾期将视为承认本报告。8）因报告中所用语言产生的歧义，以中文为准。

# 检测报告

（报告编号#WA2022025051）　　发布日期:2022年08月30日

样品编号 FT22012105001　　样品批号：　Z220802

**检测结果：**

| 序号 | 检测项目 | 单位 | 检测方法 | 检测结果 | 标准要求 | 结果判定 |
|---|---|---|---|---|---|---|
| 1 | 菌落总数 | CFU/g | ZC111 | <10 | ≤1000 | 符合标准 |
| 2 | 大肠菌群 | MPN/g | ZC112 | <0.30 | ≤0.92 | 符合标准 |
| 3 | 霉菌和酵母 | CFU/g | ZC0201 | <10 | ≤50 | 符合标准 |
| 4 | 金黄色葡萄球菌 | /25g | ZC179 | 0 | ≤0 | 符合标准 |
| 5 | 沙门氏菌 | /25g | ZC178 | 0 | ≤0 | 符合标准 |

**备注：**　无

**结果说明：**　无

捷通（广州）检测技术服务有限公司
（检验检测专用章）

***结束***


中国·广东·广州市黄埔区石英路4号27号楼、17号楼一层全层　邮编：510730　投诉热线：(86-20)-85195770

AGTC

**检测报告** （报告编号#WA2022025052） 发布日期:2022年08月30日

| | |
|---|---|
| 客户名称: | 安利（中国）研发中心有限公司 |
| 客户地址: | 广州经济技术开发区北围工业区一区临江路 1 号 |
| 样品名称: | PCA |
| 生产商/供应商: | ACCL |
| 样品批号: | （见下页） |
| 生产日期: | / |

**以上样品及信息由客户提供及确认，AGTC不承担证实客户提供信息的准确性，适当性和（或）完整性责任。**

| | |
|---|---|
| 委托单号: | WA2022025052 |
| 样品接收日期: | 2022年08月23日 |
| 样品状态: | 包装完好。 |
| 检测周期: | 2022年08月23日-2022年08月29日 |
| 检测要求: | 根据客户提供的10134的第5版文件检测。 |
| 检测方法: | （见下页） |
| 检测结果: | （见下页） |

**除非另有说明，本检测结果仅与被检测物品有关。仅供客户内部使用，不对社会具有证明作用。未经检验机构书面同意，委托人不得擅自使用检测结果进行不当宣传。**

捷通（广州）检测技术服务有限公司
（检验检测专用章）

AGTC 授权签字人

**声明：**1）捷通（广州）检测技术服务有限公司，简称 ：AGTC。 2）报告未加盖本公司检验检测专用章无效，无相关授权人签字无效。 3）未经本公司书面批准不得部分复制报告，全部复制除外。 4）报告涂改增删无效。5）对送检样品，报告仅对收到的样品负责。6）未经本公司允许，任何人不得使用本报告进行不当宣传。7）对报告的异议应于报告签发之日起15日内向本公司提出，逾期将视为承认本报告。8）因报告中所用语言产生的歧义，以中文为准。


中国•广东•广州市黄埔区石英路4号27号楼、17号楼一层全层 邮编：510730 投诉热线：（86-20）-85195770

**检测报告**　　（报告编号#WA2022025052）　　发布日期:2022年08月30日

样品编号 FT22012106001　　样品批号：　Z220801

**检测结果：**

| 序号 | 检测项目 | 单位 | 检测方法 | 检测结果 | 标准要求 | 结果判定 |
|---|---|---|---|---|---|---|
| 1 | 菌落总数 | CFU/g | ZC111 | <10 | ≤1000 | 符合标准 |
| 2 | 大肠菌群 | MPN/g | ZC112 | <0.30 | ≤0.92 | 符合标准 |
| 3 | 霉菌和酵母 | CFU/g | ZC0201 | <10 | ≤50 | 符合标准 |
| 4 | 金黄色葡萄球菌 | /25g | ZC179 | 0 | ≤0 | 符合标准 |
| 5 | 沙门氏菌 | /25g | ZC178 | 0 | ≤0 | 符合标准 |

**备注：**　无

**结果说明：**　无

捷通（广州）检测技术服务有限公司
（检验检测专用章）

***结束***


中国・广东・广州市黄埔区石英路4号27号楼、17号楼一层全层　邮编：510730　投诉热线：(86-20)-85195770
