# Supplementary material for: Effects of Protocatechuic Acid on Endothelial Function and HDL Functionality in Hypercholesterolemic Subjects: A Randomized Double-Blind Placebo-Controlled Trial
Source: Nutrients. 2026 Sep 18;18(18):3056. doi: 10.3390/nu18183056 (PMC13610876; doi:10.3390/nu18183056)
Supplement: Supplementary file 1 [file nutrients-18-03056-s001.zip › Supplementary Information S2. Translated PCA Safety.pdf]

AGTC

Test Report (Report No. #WA2022025051) Issue Date: August 30, 2022

Client Name: Amway (China) R&D Center Co., Ltd.

Client Address: Block 1, Beiwei Industrial Zone, North Linjiang Road, Guangzhou Economic- Technological Development Zone

Sample Name: / PCA

Manufacturer/Supplier: ACCL

Sample Batch No.: (See next page)

Production Date: N/A

The above- mentioned samples and information are provided and confirmed by the client. AGTC shall not be responsible for verifying the accuracy, appropriateness and/or completeness of the information provided by the client.

Work Order No.: WA2022025051

Sample Receipt Date: August 23, 2022

Sample Condition: Intact packaging.

Test Period: August 23, 2022 – August 29, 2022

Test Requirement: Test in accordance with Document 10134 Version 5 provided by the client.

Test Methods: (See next page)

Test Results: (See next page)

Unless otherwise stated, this test result is solely related to the tested item. It is for internal use by the customer only and does not have any proof value for society. Without the written consent of the testing institution, the client shall not use the test result for improper publicity without authorization.

Disclaimer:

1) Jietong (Guangzhou) Inspection & Testing Service Co., Ltd., abbreviated as AGTC.

2) This report is invalid without the company's special inspection- and- testing seal and valid authorized- signatory signature.

3) Partial reproduction of this report is prohibited without written approval from AGTC; full reproduction is permitted.

4) Tampered or altered reports are invalid.

5) For submitted samples, test results apply only to the samples received.

6) No person may use this report for improper publicity without AGTC's permission.

7) Objections to this report shall be submitted to AGTC within 15 calendar days from the report- issuance date; failure to submit within this period constitutes acceptance of the report.

8) In case of linguistic ambiguity in this report, the Chinese version shall prevail.

AGTC

Test Report (Report No. #WA2022025051) Issue Date: August 30, 2022

Sample ID: FT22012105001 Sample Batch No.: Z220802

Test Results

No.	Test Item	Unit	Test Method	Test Result	Specification Requirement	Evaluation
1	Aerobic plate count	CFU/g	ZC111	<10	≤ 1000	Compliant
2	Coliforms	MPN/g	ZC112	<0.30	≤ 0.92	Compliant
3	Molds and yeasts	CFU/g	ZC0201	<10	≤ 50	Compliant
4	Staphylococcus aureus	/25 g	ZC179	0	≤ 0	Compliant
5	Salmonella	/25 g	ZC178	0	≤ 0	Compliant

Remarks: None

Result Notes: None

AGTC

Test Report (Report No. #WA2022025052) Issue Date: August 30, 2022

Client Name: Amway (China) R&D Center Co., Ltd.

Client Address: Block 1, Beiwei Industrial Zone, North Linjiang Road, Guangzhou Economic- Technological Development Zone

Sample Name: PCA

Manufacturer/Supplier: ACCL

Sample Batch No.: (See next page)

Production Date: N/A

The above- mentioned samples and information are provided and confirmed by the client. AGTC shall not be responsible for verifying the accuracy, appropriateness and/or completeness of the information provided by the client.

Work Order No.: WA2022025052

Sample Receipt Date: August 23, 2022

Sample Condition: Intact packaging.

Test Period: August 23, 2022 – August 29, 2022

Test Requirement: Test in accordance with Document 10134 Version 5 provided by the client.

Test Methods: (See next page)

Test Results: (See next page)

Unless otherwise stated, this test result is solely related to the tested item. It is for internal use by the customer only and does not have any proof value for society. Without the written consent of the testing institution, the client shall not use the test result for improper publicity without authorization.

Disclaimer:

1) Jietong (Guangzhou) Inspection & Testing Service Co., Ltd., abbreviated as AGTC.

2) This report is invalid without the company's special inspection- and- testing seal and valid authorized- signatory signature.

3) Partial reproduction of this report is prohibited without written approval from AGTC; full reproduction is permitted.

4) Tampered or altered reports are invalid.

5) For submitted samples, test results apply only to the samples received.

6) No person may use this report for improper publicity without AGTC's permission.

7) Objections to this report shall be submitted to AGTC within 15 calendar days from the report- issuance date; failure to submit within this period constitutes acceptance of the report.

8) In case of linguistic ambiguity in this report, the Chinese version shall prevail.

AGTC

Test Report (Report No. #WA2022025052) Issue Date: August 30, 2022

Sample ID: FT22012106001 Sample Batch No.: Z220801

Test Results

No.	Test Item	Unit	Test Method	Test Result	Specification Requirement	Evaluation
1	Aerobic plate count	CFU/g	ZC111	<10	≤ 1000	Compliant
2	Coliforms	MPN/g	ZC112	<0.30	≤ 0.92	Compliant
3	Molds and yeasts	CFU/g	ZC0201	<10	≤ 50	Compliant
4	Staphylococcus aureus	/25 g	ZC179	0	≤ 0	Compliant
5	Salmonella	/25 g	ZC178	0	≤ 0	Compliant

Remarks: None

Result Notes: None

End of Report
