# Supplementary material for: Effects of Protocatechuic Acid on Endothelial Function and HDL Functionality in Hypercholesterolemic Subjects: A Randomized Double-Blind Placebo-Controlled Trial
Source: Nutrients. 2026 Sep 18;18(18):3056. doi: 10.3390/nu18183056 (PMC13610876; doi:10.3390/nu18183056)
Supplement: Supplementary file 1 [file nutrients-18-03056-s001.zip › Supplementary Information S3. PCA.pdf]

西安通泽生物科技有限公司
Xi'An TongZe BiotechCo.,Ltd

质量报告单

产品名称<br>**Product Name**	（**3,4-**二羟基苯甲酸）原儿茶酸<br>**3,4-l）>hy<lroxybenzoic Acid**		
**CAS** 号 **CAS No.**	**99-50-3**	产品批号<br>**Batch Number**	**TZ20220703-1**
包装规格<br>**Printed Box**	**40kg/**袋 **xl** 袋/桶	生产日期<br>**Manufacture Date**	**2022.07.03**
检测日期<br>**Test Date**	**2022.07.05**	有效期至<br>**Expiry Date**	**2024.07.03**
分析项目 **Items of Analysis**	质量标准<br>**Specification**		实测结果<br>**Results**
外观<br>**Appearance**	白色至类白色结晶粉末<br>**White or almost white crystalline powder**		符合规定 **Complies**
原儿茶酸含量<br>**Assay**	**>99.0%**		**99.63%**
溶解性<br>**Solubility**	溶于热水<br>**Soluble in hot water**		符合规定<br>**Complies**
水分<br>**Moisture**	不高于 **0.5%**<br>**not more than 0.5%**		**0.37%**
单杂<br>**Any individual impurity**	不高于 **0.2%**<br>**not more than 0.2%**		符合规. 定<br>**Complies**
残留溶剂<br>i **Residual solvents**	不得检出<br>**Must not be checked out**		符合规定
判定<br>**Decide**	本品符合 **Q/VBL 0002S-2021** 之，**This product complies with Q/VBL 0002^2024^4^**		

检验人 Analyst: 李力

复核人 Corrector: 王娜
