# Supplementary material for: Effects of Protocatechuic Acid on Endothelial Function and HDL Functionality in Hypercholesterolemic Subjects: A Randomized Double-Blind Placebo-Controlled Trial
Source: Nutrients. 2026 Sep 18;18(18):3056. doi: 10.3390/nu18183056 (PMC13610876; doi:10.3390/nu18183056)
Supplement: Supplementary file 1 [file nutrients-18-03056-s001.zip › Supplementary Information S4. Translated PCA.pdf]

Quality Report

Product Name	Protocatechuic Acid (3,4-dihydroxybenzoic acid)		
CAS No.	99-50-3	Batch Number	TZ20220703-1
Printed Box	40 kg/bag, 1 bag per drum	Manufacture Date	July 03, 2022
Test Date	July 05, 2022	Expiry Date	July 03, 2024
Items of Analysis	Specification		Test Results
Appearance	White or almost-white crystalline powder		Complies
Purity	>99.0 %		99.63 %
Solubility	Soluble in hot water		Complies
Moisture	Not more than 0.5 %		0.37 %
Any individual impurity	Not more than 0.2 %		Complies
Residual solvents	Must not be detected		Complies
Decide	This product complies with enterprise standard Q/VBL 0002S-2021.		
