# Supplementary material for: Effects of Protocatechuic Acid on Endothelial Function and HDL Functionality in Hypercholesterolemic Subjects: A Randomized Double-Blind Placebo-Controlled Trial
Source: Nutrients. 2026 Sep 18;18(18):3056. doi: 10.3390/nu18183056 (PMC13610876; doi:10.3390/nu18183056)
Supplement: Supplementary file 1 [file nutrients-18-03056-s001.zip › Supplementary Tables and Figures.pdf]

Table S1. Mean daily intake of nutrients of study population[1]

		Baseline	6 weeks	12 weeks	*P* value[2]
Energy, kcal/d	Placebo	2346.84 (2300.39 to 2393.28)	2337.39 (2296.57 to 2378.20)	2327.22 (2281.40 to 2373.05)	0.653
	PCA	2349.08 (2310.79 to 2387.37)	2290.36 (2245.57 to 2335.16)	2323.62 (2285.55 to 2361.69)	
Protein, g/d	Placebo	89.32 (87.06 to 91.59)	88.21 (85.76 to 90.67)	89.23 (87.33 to 91.14)	0.417
	PCA	90.61 (88.05 to 93.18)	91.21 (88.82 to 93.59)	89.00 (86.81 to 91.20)	
Total carbohydrate, g/d	Placebo	304.26 (294.46 to 314.07)	301.90 (293.37 to 310.44)	298.64 (288.75 to 308.53)	0.977
	PCA	303.03 (294.87 to 311.19)	289.36 (279.35 to 299.37)	300.62 (292.08 to 309.16)	
Total fat, g/d	Placebo	85.64 (83.16 to 88.12)	86.32 (83.97 to 88.68)	86.19 (84.10 to 88.29)	0.737
	PCA	86.19 (83.86 to 88.52)	85.35 (83.09 to 87.60)	85.01 (82.86 to 87.17)	
Cholesterol, mg/d	Placebo	295.97 (284.96 to 306.98)	312.66 (301.05 to 324.27)	299.48 (287.30 to 311.67)	0.437
	PCA	302.51 (290.75 to 314.26)	294.46 (284.17 to 304.76)	307.48 (297.57 to 317.38)	
Fiber, g/d	Placebo	19.25 (17.53 to 20.97)	19.02 (17.29 to 20.74)	18.35 (16.71 to 20.00)	0.183
	PCA	17.95 (15.99 to 19.91)	20.79 (18.82 to 22.75)	19.01 (17.40 to 20.63)	

[1]All values are means (95% CI). [2]A linear mixed model of repeated measures with terms for treatment, time, and time × treatment interactions as fixed effects with an unstructured residual covariance matrix across visits and no random effects; and baseline values, age and sex as covariates, *P* value for the overall treatment fixed effect. $P < 0.05$ indicates a significant difference. CI, confidence interval; PCA, protocatechuic acid.

Table S2. Changes in cardio-metabolic biomarkers of study population[1]

	6-week change[2]	12-week change[2]	Adjusted PCA-Placebo Difference at week 12	*P* value (12 week)[3]
TC, mmol/L				
Placebo	-0.02 (-0.26 to 0.21)	0.08 (-0.15 to 0.30)		
PCA	0.07 (-0.17 to 0.31)	-0.11 (-0.36 to 0.14)	-0.07 (-0.26 to 0.13)	0.489
TG, mmol/L				
Placebo	-0.03 (-0.12 to 0.07)	-0.05 (-0.14 to 0.05)		
PCA	0.02 (-0.07 to 0.11)	0.02 (-0.09 to 0.14)	-0.03 (-0.10 to 0.04)	0.402
HDL-C, mmol/L				
Placebo	0.10 (-0.04 to 0.24)	0.08 (-0.06 to 0.22)		
PCA	-0.07 (-0.21 to 0.07)	-0.06 (-0.22 to 0.10)	-0.04 (-0.12 to 0.03)	0.290
LDL-C, mmol/L				
Placebo	0.06 (-0.07 to 0.18)	-0.02 (-0.15 to 0.10)		
PCA	0.03 (-0.10 to 0.15)	-0.06 (-0.19 to 0.06)	-0.02 (-0.11 to 0.07)	0.727
Apo AⅠ, g/L				
Placebo	0.02 (-0.14 to 0.17)	0.05 (-0.09 to 0.19)		
PCA	-0.02 (-0.14 to 0.10)	0.06 (-0.06 to 0.19)	-0.07 (-0.16 to 0.01)	0.097
Apo B, g/L				
Placebo	-0.07 (-0.19 to 0.05)	0.00 (-0.14 to 0.14)		
PCA	-0.05 (-0.18 to 0.07)	-0.02 (-0.14 to 0.10)	0.03 (-0.06 to 0.12)	0.533
7-KC, nmol/L				
Placebo	0.76 (0.57 to 0.96)	0.05 (-0.13 to 0.23)		
PCA	0.41 (-0.81 to 1.63)	-0.67 (-1.91 to 0.58)	-0.41 (-1.25 to 0.44)	0.346
Glucose, mmol/L				

Table S2 (Continued).

Placebo	-0.05 (-0.18 to 0.08)	-0.07 (-0.19 to 0.06)		
PCA	0.06 (-0.08 to 0.20)	0.07 (-0.04 to 0.19)	0.04 (-0.05 to 0.13)	0.340
Insulin, μU/L				
Placebo	-0.47 (-1.50 to 0.55)	-0.36 (-1.42 to 0.69)		
PCA	0.02 (-1.07 to 1.11)	0.68 (-0.42 to 1.78)	-0.01 (-0.80 to 0.79)	0.986
HOMA-IR				
Placebo	-0.12 (-0.35 to 0.10)	-0.11 (-0.35 to 0.14)		
PCA	0.01 (-0.22 to 0.23)	0.19 (-0.06 to 0.45)	0.02 (-0.15 to 0.20)	0.797
SBP, mmHg				
Placebo	0.34 (-1.56 to 2.23)	1.49 (-0.36 to 3.34)		
PCA	-1.20 (-2.56 to 0.16)	-7.35 (-8.76 to -5.94)	-8.24 (-10.22 to -6.26)	<0.001
DBP, mmHg				
Placebo	-1.27 (-3.86 to 1.32)	1.68 (-1.31 to 4.66)		
PCA	-1.05 (-4.14 to 2.03)	0.31 (-2.11 to 2.73)	-0.28 (-2.42 to 1.86)	0.796
Heart rate, beats/min				
Placebo	0.22 (-2.00 to 2.43)	-0.46 (-2.57 to 1.65)		
PCA	-1.74 (-4.20 to 0.71)	-2.81 (-5.30 to -0.33)	-0.99 (-2.98 to 1.00)	0.329

[1]All values are means (95% CI). [2]Data are presented as raw mean (95% CI) for changes from baseline. [3]The main effect of intervention was analyzed by a linear mixed model of repeated measures with terms for treatment, time, and time × treatment interactions as fixed effects with an unstructured residual covariance matrix across visits and no random effects; and baseline values, age and sex as covariates, *P* value for the week-12 contrast. $P < 0.05$ indicates a significant difference. 7-KC, 7-ketocholesterol; Apo AI, apolipoprotein AI; Apo B, apolipoprotein B; CI, confidence interval; DBP, diastolic blood pressure; HDL-C, high-density lipoprotein cholesterol; HOMA-IR, homeostasis model assessment of insulin resistance; LDL-C, low-density lipoprotein cholesterol; PCA, protocatechuic acid; SBP, systolic blood pressure; TC, total cholesterol; TG, triglyceride.

Table S3. Changes in hematological indices, liver and kidney function of study population[1]

	6-week change	12-week change	*P* value[2]
Red blood cell, $\times 10^{12}$/L			0.775
Placebo	0.10 (-0.02 to 0.22)	0.08 (-0.05 to 0.20)	
PCA	0.04 (-0.12 to 0.20)	0.03 (-0.12 to 0.17)	
White blood cell, $\times 10^{9}$/L			0.607
Placebo	0.27 (-0.03 to 0.57)	-0.05 (-0.33 to 0.23)	
PCA	0.10 (-0.20 to 0.39)	0.13 (-0.22 to 0.48)	
Hemoglobin, g/L			0.402
Placebo	1.20 (-2.05 to 4.46)	-2.45 (-5.43 to 0.53)	
PCA	-0.64 (-3.48 to 2.20)	1.27 (-1.74 to 4.28)	
Creatinine, µmol/L			0.645
Placebo	-2.83 (-6.17 to 0.51)	-1.52 (-4.91 to 1.86)	
PCA	-1.22 (-4.09 to 1.66)	1.02 (-2.35 to 4.38)	
Bilirubin, µmol/L			0.258
Placebo	-0.78 (-1.88 to 0.33)	1.05 (0.14 to 1.95)	
PCA	0.48 (-0.42 to 1.38)	-1.34 (-2.48 to -0.23)	
Urea, mmol/L			0.253
Placebo	0.96 (0.40 to 1.52)	0.19 (-0.49 to 0.87)	
PCA	-0.04 (-0.68 to 0.60)	1.05 (0.41 to 1.69)	
Total protein, g/L			0.595
Placebo	0.42 (-1.22 to 2.07)	0.45 (-0.95 to 1.85)	
PCA	0.25 (-1.22 to 1.72)	0.01 (-1.04 to 1.06)	
Albumin, g/L			0.620

Table S3 (Continued).

Placebo	-0.19 (-1.22 to 0.85)	-0.11 (-1.18 to 0.97)	
PCA	-0.20 (-1.31 to 0.91)	0.23 (-0.94 to 1.41)	
AST, U/L			0.758
Placebo	-2.69 (-4.72 to -0.66)	-1.40 (-3.28 to 0.48)	
PCA	-1.54 (-3.55 to 0.46)	0.39 (-1.25 to 2.04)	
ALT, U/L			0.438
Placebo	-1.73 (-4.60 to 1.13)	2.07 (-0.96 to 5.10)	
PCA	2.05 (-0.70 to 4.80)	-2.22 (-5.48 to 1.04)	

[1]All values are means (95% CI). [2]The main effect of intervention was analyzed by a linear mixed model of repeated measures with terms for treatment, time, and time × treatment interactions as fixed effects with an unstructured residual covariance matrix across visits and no random effects; and baseline values, age and sex as covariates, *P* value for the overall treatment fixed effect. $P < 0.05$ indicates a significant difference. ALT, alanine aminotransferase; AST, aspartate transaminase; CI, confidence interval; PCA, protocatechuic acid.

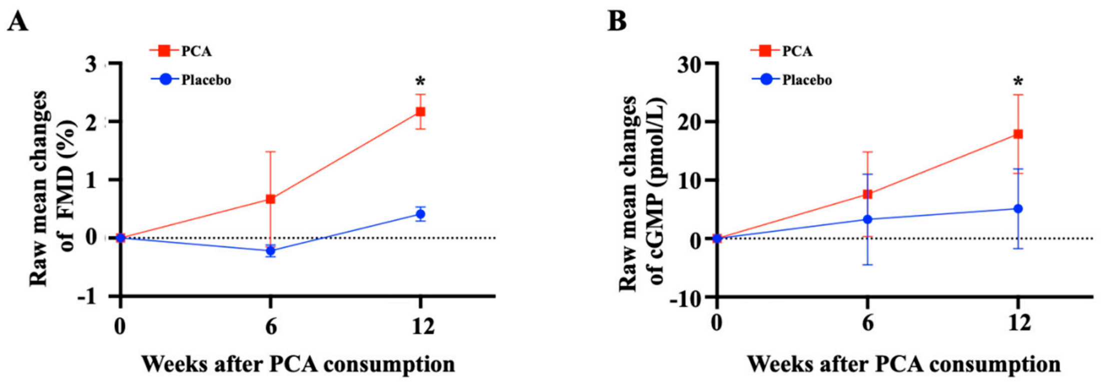

Supplementary Figure S1. Beneficial effect of PCA on endothelial function in participants with hypercholesterolemia. (A, B) raw mean changes in (A) FMD and (B) cGMP and two-sided 95% CIs for each group and the pairwise comparisons between two groups, from baseline to week 12, respectively. Results are from the linear mixed effects models with repeated measures including 148 participants who completed the trial. *$P < 0.05$ indicates a significant difference between PCA and placebo. cGMP, cyclic guanosine monophosphate; FMD, flow-mediated dilation; PCA, protocatechuic acid.

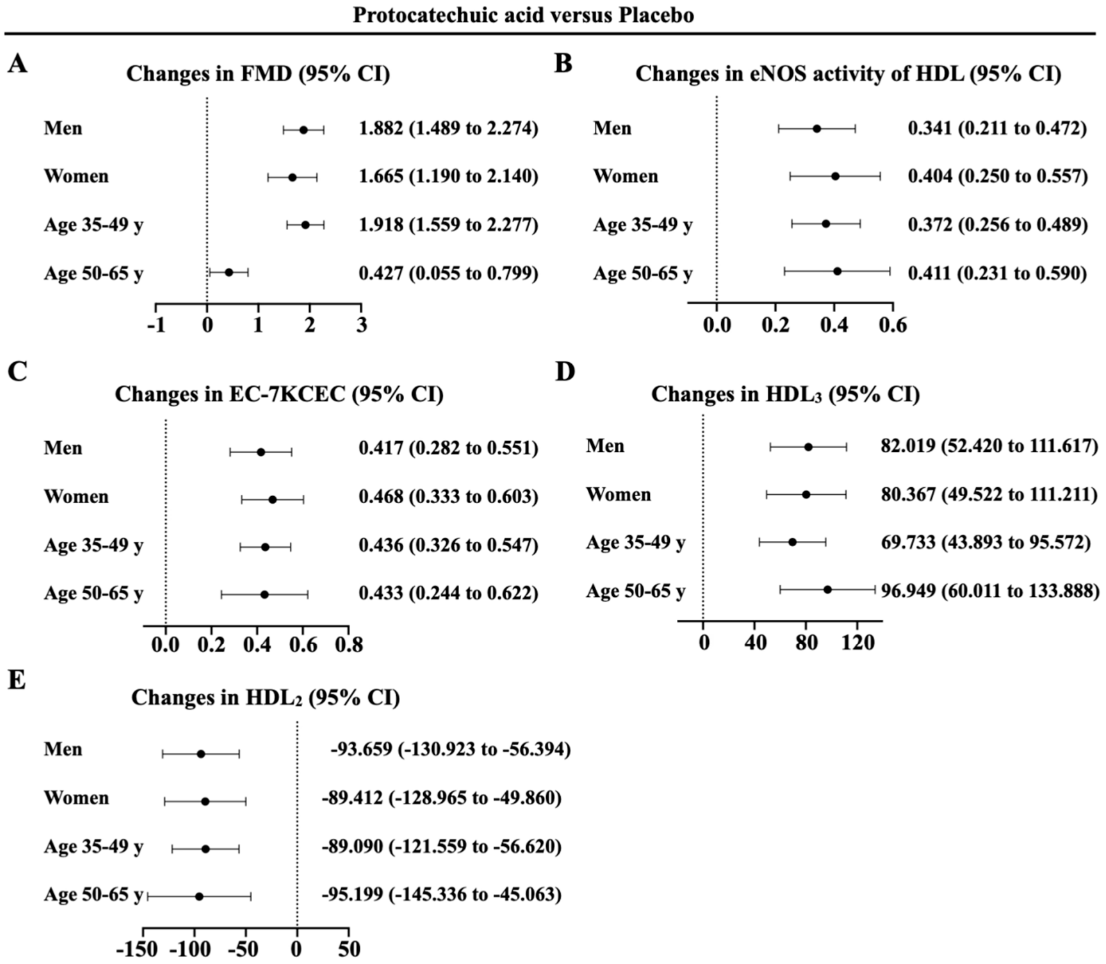

Supplementary Figure S2. Changes in the outcomes of endothelial function and HDL subclass distribution after a 12-week intervention between PCA and placebo. Mean changes in FMD (A), eNOS activity of HDL (B), EC-7KCEC (C), HDL_3 concentration (D) and HDL_2 concentration (E) by sex and age groups after a 12-week intervention between PCA and Placebo. Results are from the linear mixed effects models with repeated measures including 148 participants who completed the trial. CI, confidence interval; EC-7KCEC, cultured human aortic endothelial 7-ketocholesterol efflux capacity; eNOS, endothelial nitric oxide synthase; FMD, flow-mediated dilation; HDL, high-density lipoprotein cholesterol.
